# Circadian behaviour of *Tectus* (*Trochus*) *niloticus* in the southwest Pacific inferred from accelerometry

**DOI:** 10.1186/s40462-015-0054-5

**Published:** 2015-09-16

**Authors:** Aurélie Jolivet, Laurent Chauvaud, Julien Thébault, Anthony A. Robson, Pascal Dumas, George Amos, Anne Lorrain

**Affiliations:** Institut Universitaire Européen de la Mer (UMR CNRS 6539), Université de Bretagne Occidentale, rue Dumont d’Urville, F-29280 Plouzané, France; LabexMER, UMS 3113 CNRS, Institut Universitaire Européen de la Mer, Université de Brest, Rue Dumont D’Urville, 29280 Plouzané, France; Atmosphere and Ocean Research Institute, The University of Tokyo, 5-1-5, Kashiwanoha, Kashiwa, Chiba 277-8564 Japan; Institut de Recherche pour le Développement (UR 227 CoReUs), 2, Fisheries Department of Vanuatu, Port-Vila, Vanuatu; SANMA Fisheries Department of Vanuatu, Port-Vila, Vanuatu; Institut de Recherche pour le Développement (UMR 6539 CNRS/UBO/IRD/IFREMER), BPA5, 98848 Nouméa cedex, Nouvelle Calédonie

**Keywords:** Activity, Accelerometry, 24-hour periodicity, *Tectus niloticus*, Foraging, Migration

## Abstract

**Background:**

Behaviour and time spent active and inactive are key factors in animal ecology, with important consequences for bioenergetics. For the first time, here, we equipped the gastropod *Tectus* (= *Trochus*) *niloticus* with accelerometers to describe activity rhythms at two sites in the Southwest Pacific with different temperature regimes: New Caledonia and Vanuatu.

**Results:**

Based on a 24-hour cycle, *T. niloticus* activity began at dusk and gradually stopped during the night, before sunrise. This nocturnal behaviour was characterised by short (duration <30 s), low intensity (acceleration < 0.12 *ɡ*) movements and was probably associated with foraging behaviour. We assumed that activity ceased once the animal was satiated. Our analysis of two size groups in Vanuatu (80–90 mm vs. 120–140 mm, basal shell diameter) revealed a size effect; smaller specimens displayed greater activity, reflected by more intense and longer movements while migrating at night toward the edge of the reef. This nocturnal behaviour is not uncommon for grazing gastropods and is mainly associated with attempting to avoid visual predators whilst feeding.

**Conclusions:**

The use of accelerometers coupled with light and temperature sensors provided detailed information on topshell behaviour and physiology under natural conditions. These data provide a foundation for identifying potential changes in the fine-scale behaviour of *T. niloticus* in response to environmental changes, which is essential in animal ecology and stock conservation.

## Background

Behaviour is a fundamental part of the biology of organisms; it is a manifestation of the response of an individual to its environment and provides a connection to the organism’s physiological condition [1, 2]. Understanding and quantifying animal behaviour has important implications for conservation, particularly when combined with knowledge on space usage, since appropriate localities often need to be protected. Generally, behavioural descriptions have been obtained through direct observations that are limited to a small fraction of an animal’s daily activities; however, animals could be affected by the presence of the human investigator [3–5].

Over the past few years, animal-attached technology has been increasingly used to address issues related to conservation [1, 6, 7]. Remote monitoring of animal behaviour allows investigators to locate and observe animals and to record their habits with virtually no limitations due to visibility, observer bias, or geographic scale [1, 8, 9]. Accelerometry is a valuable method for obtaining descriptions of behaviours, such as locomotion, foraging, and escaping predators, as well as exploring the influence of environmental parameters and/or energy budgets in a range of animals [10–14]. Combining this technology with others sensors on tagged animals, such as light and ambient temperature sensors, allows the examination of activity rhythms in relation to environmental conditions [15–17]. Brown et al. [18] noted that accelerometry studies between 1998 and 2012 generally focused on mammals, birds, fish or reptiles, encompassing only three molluscs: the cuttlefish *Sepia apama* [10], the giant squid *Dosidicus gigas* [16], and the great scallop *Pecten maximus* [11, 19]. To our knowledge, gastropods have not yet been investigated in this framework.

The topshell *Tectus niloticus*, formerly known as *Trochus niloticus*, has been harvested for subsistence and commercial purposes in the Indo-West Pacific [20, 21]. Overfishing of topshells has been reported in many areas [22, 23], resulting in the implementation of fisheries management -based size restrictions, closed seasons, marine sanctuaries, and restocking [24–30]. This species is distributed on outer reef flats and reef crests with abundant stony corals, on areas of turf algae for adults, and on intertidal reef flats with stony coral/rubble bottom for juveniles [28, 31–33]. *T. niloticus* is found on coral reefs from high water to 20 m depth but they are abundant at depths of <8 m [28, 34]. The highest densities of *T. niloticus* are in areas characterized by substrates with low structural complexity with a heavy cover of coral rubble, algal pavement and unobstructed exposure to surf [22, 34, 35]. These topshells are nocturnally active, grazing herbivores that hide in the holes and crevices of massive corals like Porites during daytime [21, 36, 37]. *T. niloticus* usually live for 15–20 years [38], reach reproductive maturity after two years (when they are ~ 6 cm, basal shell diameter) [21, 36, 39] and have limited dispersal capabilities [20, 39].

Although the nocturnal behaviour of topshells has already been observed [21, 36, 37], to our knowledge no quantification of their nocturnal activity has been performed. Here, we applied accelerometry technology to characterise the duration and intensity of *T. niloticus* activity in their natural environment in New Caledonia and Vanuatu. We also compared *T. niloticus* activity to metabolic measurements performed simultaneously and published in a companion paper [40].

## Methods

### Technology

The HOBO Pendant G data logger (UA-004-64, Onset Computer Corporation) is an accelerometer with the ability to measure acceleration in three axes with a range of ± 3 ɡ (29.4 m.s^−2^) at a resolution of 0.025 ɡ (0.245 m.s^−2^) and with accuracies of ± 0.075 ɡ (0.735 m.s^−2^) at 25 °C and ± 0.105 ɡ (1.03 m.s^−2^) from −20 °C to 70 °C. The data logger is packaged in waterproof housing and has dimensions of 58 × 33 × 23 mm (sectional area 7.6 cm^2^). Its memory capacity of 64 kB allows the data logger to record movements for 24 h and 13 min at a frequency of 0.25 Hz. Data were downloaded and recording was reset every 24 h using a HOBO Waterproof Shuttle (U-DTW-1, Onset Computer Corporation). The download took less than one minute per individual, and was carried out directly on the topshells without moving or manipulating them.

### Accelerometer attachment

**Fig. 1:**
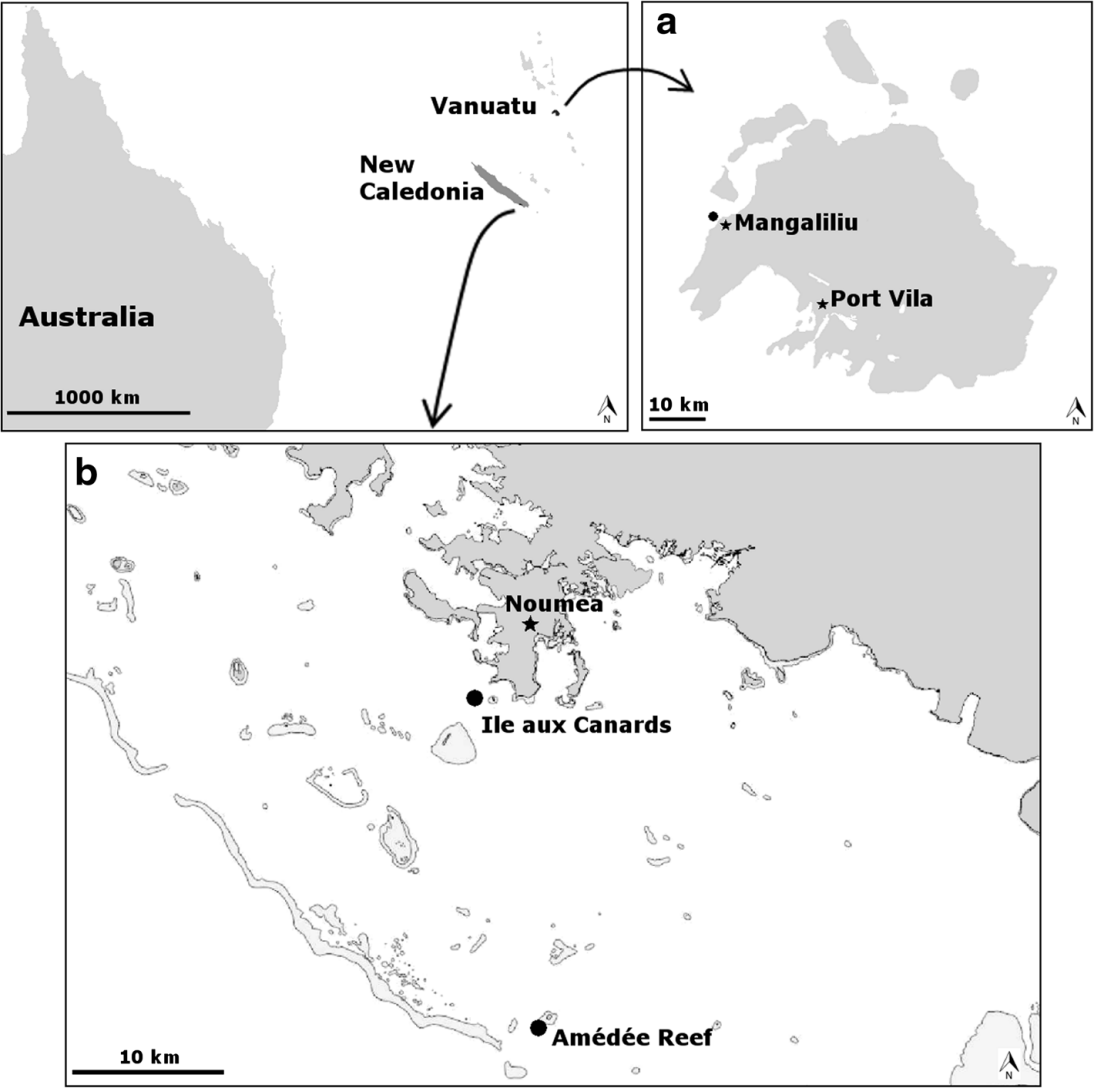
Location of study sites (black circles). **a** Vanuatu with the capture and monitoring site off Mangaliliu. **b** New Caledonia with Amédée reef (the capture site) and Ile aux Canards (the monitoring site)

**Fig. 2:**
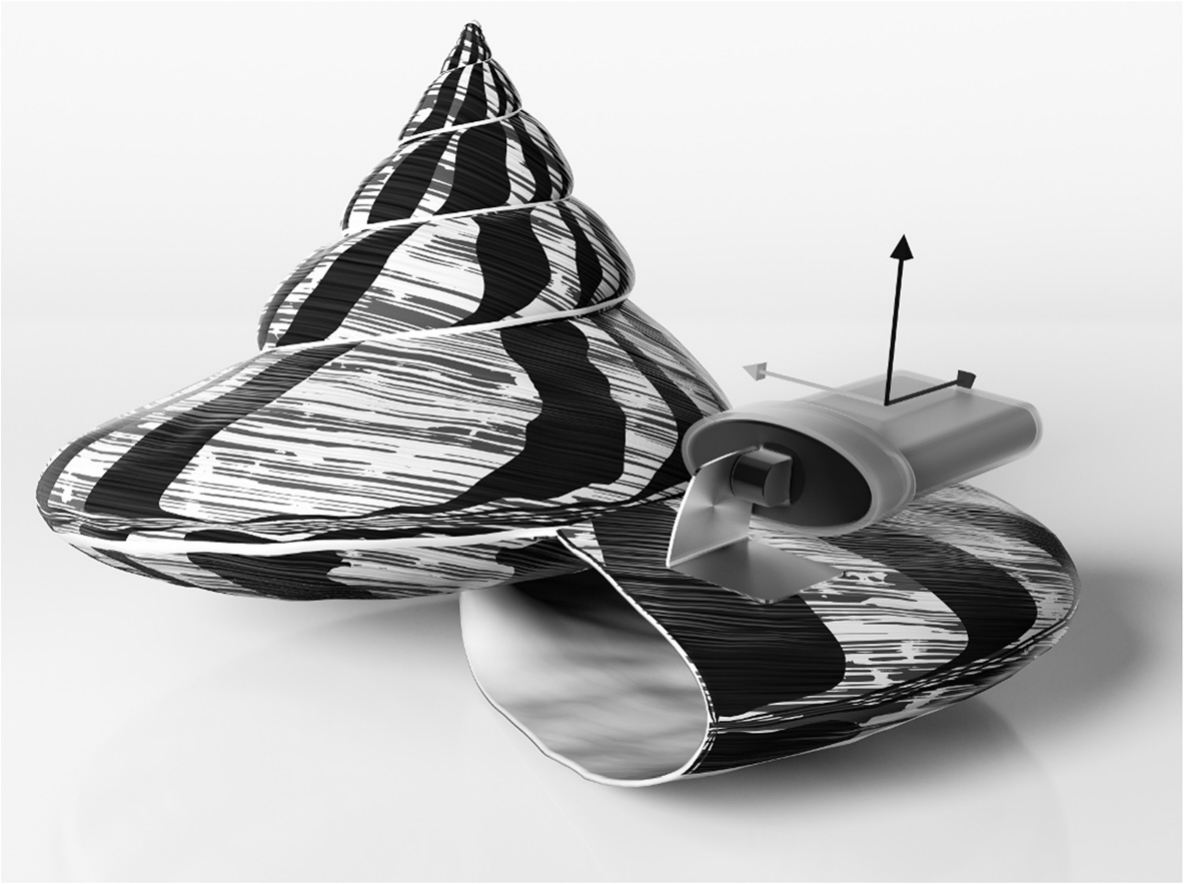
Large Trochus (B_d_ = 120 mm) instrumented with accelerometer. HOBO Pendant G data logger was attached to an aluminum tag. The arrows indicate the three measurement axes of the accelerometer

A total of 18 topshells were instrumented in New Caledonia and Vanuatu (Fig. 1). In order to attach the data logger (Fig. 2), a hole was drilled in the lower lip (back) at a distance of 1 cm from the peristomial margin. A stainless-steel, round-headed bolt was inserted in this hole (inward). The data logger was attached to an aluminium tab with a hole drilled through its middle. This system was inserted on the bolt (outside the shell) and secured with a stainless-steel nut firmly screwed onto it. To avoid injury to the topshell mantle from the bolt head, the latter was encapsulated in epoxy resin (Araldite® 90 s Fusion, Huntsman Advanced Materials). Care was taken to attach the units as similar as possible between individuals. The entire procedure required ~1 h to equip all topshells. Once instrumented, the animals were released on the monitoring site and left for 24 h without recording to avoid any tagging bias. The instrumented topshells did not appear to affect their behaviour e.g. moving or hiding in the holes and crevices.

### Deployment

Fieldwork was conducted in New Caledonia in July 2012 and in Vanuatu in June 2013, representing the cool season for each location. All *T. niloticus* specimens were harvested via scuba diving or snorkelling. Study sites were reef flats characterised by mixed substrate (slab, live coral branching, and coral debris) and shallow water (~2 m in depth). For both sites, a HOBO Waterproof Pendant data logger (UA-002-68, Onset Computer Corporation) was used to acquire temperature (°C) and relative light level (lumens m^−2^) with a frequency of one measurement per minute over the monitoring period.

In New Caledonia, 10 topshells were harvested on July 14, 2012 at Amédée reef (22°28.804 S; 166°27.877 E; Fig. 1). Basal diameters (B_d_) of only three topshells were measured (Table 1). All 10 shells were instrumented with accelerometers in the lab as described above and maintained in a seawater aquarium for 24 h before their transfer to Ile aux Canards, a coastal site (22°18.900 S; 166°26.100 E; Fig. 1). Accelerometers were started on July 16, 2012 at 15:30:00, after a 24-h acclimatisation period. Data were downloaded every day until August 1, 2012 at 09:30:00, corresponding to a 330-h of recorded data.

**Table 1:** Main characteristics (mean ± standard deviation) of topshells and activities according to site and size

	New Caledonia			Vanuatu		
	Total	Large	Small	Total	Large	Small
*Specimens*						
N _Topshell_	10	9	1	8	4	4
B_d_		120; 121.6	72.1	108.9 ± 25.9	132.3 ± 9.2	85.5 ± 5.1
*Activity (per specimen)*						
24-h	414 ± 101	335 ± 85	484 ± 94	443 ± 151	327 ± 118	560 ± 66
Night	393 ± 98	319 ± 85	449 ± 99	429 ± 145	320 ± 119	539 ± 60
Dawn	1 ± 3	0 ± 1	2 ± 2	2 ± 5	0 ± 1	4 ± 6
Day	7 ± 11	5 ± 7	15 ± 13	9 ± 13	5 ± 4	14 ± 17
Dusk	13 ± 6	10 ± 5	17 ± 7	1 ± 2	2 ± 3	1 ± 2
Movement duration (min)						
D/ 24-hour	131 ± 50	89 ± 25	188 ± 54	178 ± 103	88 ± 38	269 ± 53

Specimens were defined by number (N_Topshell_) and B_d_ (mm). Specimens are considered as small when B_d_ < 100 mm or as large when B_d_ > 100 mm. Activities were defined as the average number of movements per individual

In Vanuatu, four large and four small topshells were equipped with accelerometers (Table 1). They were harvested on June 3, 2014 off Mangaliliu (17°38.248 S; 168°12.034 E; Fig. 1) and released 1 h later, equipped with accelerometers, 10 m away from the capture site. Recording began on June 4, 2013 at 12:30:00 and was stopped June 7, 2013 at 11:25:00, corresponding to a 71-h recording period.

### Data analysis

Data from the accelerometers were downloaded onto a personal computer using HOBOware ® Pro 3.0.0. Acceleration values were given in units of ɡ, where ɡ represents acceleration due to gravity (1 ɡ = 9.81 m s^−2^).

Under static circumstances, such as during rest or after death or in the case of any absence of movement caused by the environment, accelerometer signals only represent the gravitational force acting on the sensors. When an animal is moving, sensor output represents acceleration due to gravity plus the inertial acceleration generated by movement. An approximation of absolute acceleration (in ɡ) resulting only from dynamic acceleration in each dimension was extracted from each axis following removal of static acceleration using a running mean over 25 points corresponding to 1.5 min [41]. A derivative of dynamic body acceleration, the vector sum of dynamic body acceleration (VeDBA, in ɡ), was calculated from tri-axial acceleration data as VeDBA = √ (ax^2^ + ay^2^ + az^2^) [42–45]. Here, ax, ay and az were dynamic acceleration values derived from raw x,y,z acceleration data [42]. These data represented the acceleration recorded by the data logger owing to the dynamic movement of that individual [41, 43]. Acceleration data were then processed using the packages FullDBA and BeFeatures within BEnergetix in the free R software [19].

After data processing, each identified movement was characterised by its duration D (in seconds and minutes), the average acceleration VeDBA _mean_ (in ɡ) and the total acceleration VeDBA_tot_ (VeDBA_tot_ = VeDBA_mean_ x D). For each analysed specimen, these data were compiled in 30-min intervals (a time slot of 00:00 corresponded to movement that occurred between 00:00:00 and 00:29:59) by summing the movement data within each interval (denoted by the subscript act), or by averaging these data to characterise movements performed over this period (denoted by the subscript mov). The obtained parameters were: *N* = number of movements made in the interval; D_act_ = cumulative duration of movement; D_mov_ = average duration of movements; VeDBA_act_ = average acceleration of activity during the interval (VeDBA _act_ (in ɡ) = (Σ VeDBA_tot_)/30 min); and VeDBA_mov_ = average acceleration of movements performed during the interval.

These parameters were then analysed in terms of study sites, size classes (small with B_d_ < 100 mm and large with B_d_ > 100 mm) and periods of interest (such as 24-h cycles). For each group considered, the mean was calculated and expressed ± standard deviation. To calculate the frequency of events, an autocorrelation test was first performed for each variable to ensure that the data were not randomly distributed. Then, spectral analysis was performed with the Lomb-Scargle periodogram, a cosine-based approach that estimates the period and strength (power) of multiple periodic components in time-series data [46, 47]. Spectral peaks with *p* > 0.001 or corresponding to harmonic frequencies (periods of 6 h, 8 h or 12 h for a main 24-h period) were not considered. Student’s t-tests and paired t-tests were used to test differences between measured means. The analysis of a size effect on the activity in New Caledonia was performed with three individuals (two large and one small). To estimate the potential influence of different temperatures, VeDBA_mean_ from all tagged specimens and across the entire sampling period were averaged for every 0.1 °C increment of water temperature. Activity data suggested a Gaussian distribution, thus the method proposed by Gannon et al. [48] was applied to fit a thermal performance curve for each site and each size-class, using minimum least-squares non-linear regression:

$$ VeDB{A}_{mean}=S\times {e}^{-{\left(\frac{T-{T}_{opt}}{2\sigma}\right)}^2} $$

Where S is a scalar, T_opt_ is the temperature (in °C) at which VeDBA is maximised, σ is the standard deviation for the normally distributed curve.

## Results

### Environmental parameters

**Fig. 3:**
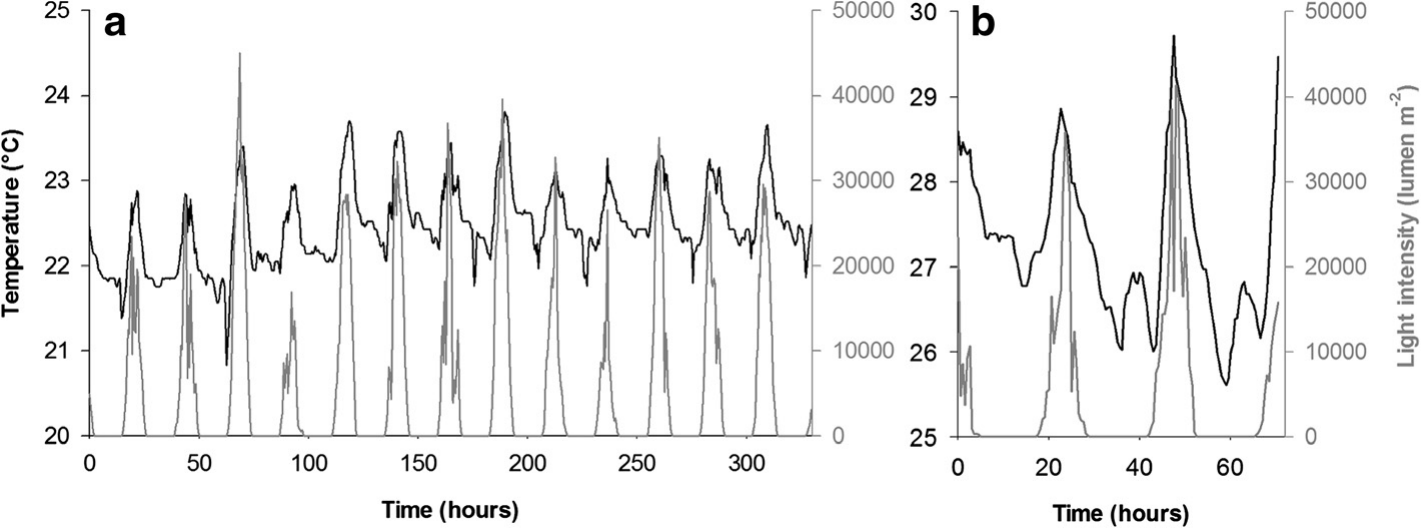
Temperatures (dark lines) and light intensities (grey lines). Measurements were taken at Ile aux Canards in New Caledonia (**a**) and off Mangalilu in Vanuatu (**b**)

**Fig. 4:**
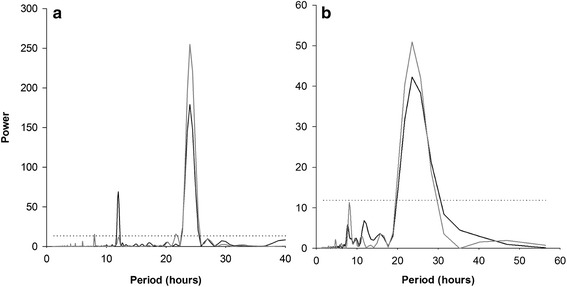
Mean power spectra (Lomb–Scargle procedure) for light intensity and number of identified movements. Light intensity (black lines) and movements (grey lines) were summed for 10 topshells over 30-min interval in New Caledonia (**a**) and Vanuatu (**b**). Dotted lines, *p* < 0.001. The main spectral peak corresponded to a period of 23.15 h, and secondary spectral peaks to harmonic frequencies at 8 h and 12 h

Of the two study sites, Vanuatu had warmer temperatures (27.3 ± 0.9 °C in Vanuatu vs. 22.5 ± 0.5 °C in New Caledonia; Fig. 3), but both sites received high intensities of light (maximum: 41 334 lumens m^−2^ in Vanuatu vs. 44 996 lumens m^−2^ in New Caledonia; Fig. 3). Four periods of light intensity were defined: day (>0 lumen m^−2^), night (0 lumen m^−2^), dawn and dusk. These last two periods corresponded to the first and last 30-min intervals with light levels > 0 lumen m^−2^ (New Caledonia: dawn at 6:00, 14.3 ± 8.5 lumens m^−2^; dusk at 17:30, 21.2 ± 11.8 lumens m^−2^. Vanuatu: dawn at 6:00, 29.7 ± 9.7 lumens m^−2^; dusk at 17:00, 55.9 ± 30.4 lumens m^−2^). Light and temperature were cyclical, with periods of 24 h for both sites (Fig. 4).

### Topshell movements

We identified 52 977 and 10 638 movements in New Caledonia and Vanuatu, respectively (Table 1). Overall, the characteristics of these data were similar between sites: D_mov_ ranged from 4 s (acquisition frequency) to 10 min, with average acceleration VeDBA_mov_ of 0.017 - 0.75 ɡ. Eighty-three percent of movements lasted < 30 s, with VeDBA_mov_ of 0.08 ± 0.03 ɡ.

### Topshell activity

**Fig. 5:**
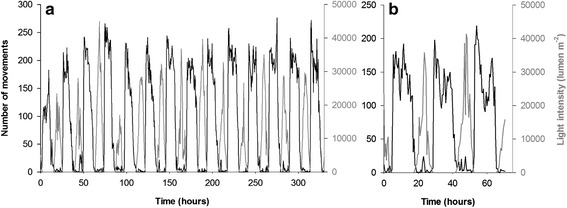
Movement numbers for all observed topshells per 30-min interval. Movements (black lines) and light intensity (grey lines) were measured in New Caledonia (**a**) and in Vanuatu (**b**)

*T. niloticus* from the two sites had comparable activity making an average of 419 ± 110 movements per day (T-test, *P* = 0.17), corresponding to 138 ± 63 min of activity (T-test, *P* = 0.08, Table 1). This activity exhibited a significant periodicity of 24 h (*p* < 0.001; Fig. 4) at both sites, but was inversely correlated with light intensity (Pearson’s correlation coefficients, *R* = −0.54 for New Caledonia, *R* = −0.57 for Vanuatu; *p* < 0.001; Fig. 5). Thus, 95 % of the movements were performed at night, 2 % during the day, 3 % at dusk, and none at dawn (Table 1).

**Fig. 6:**
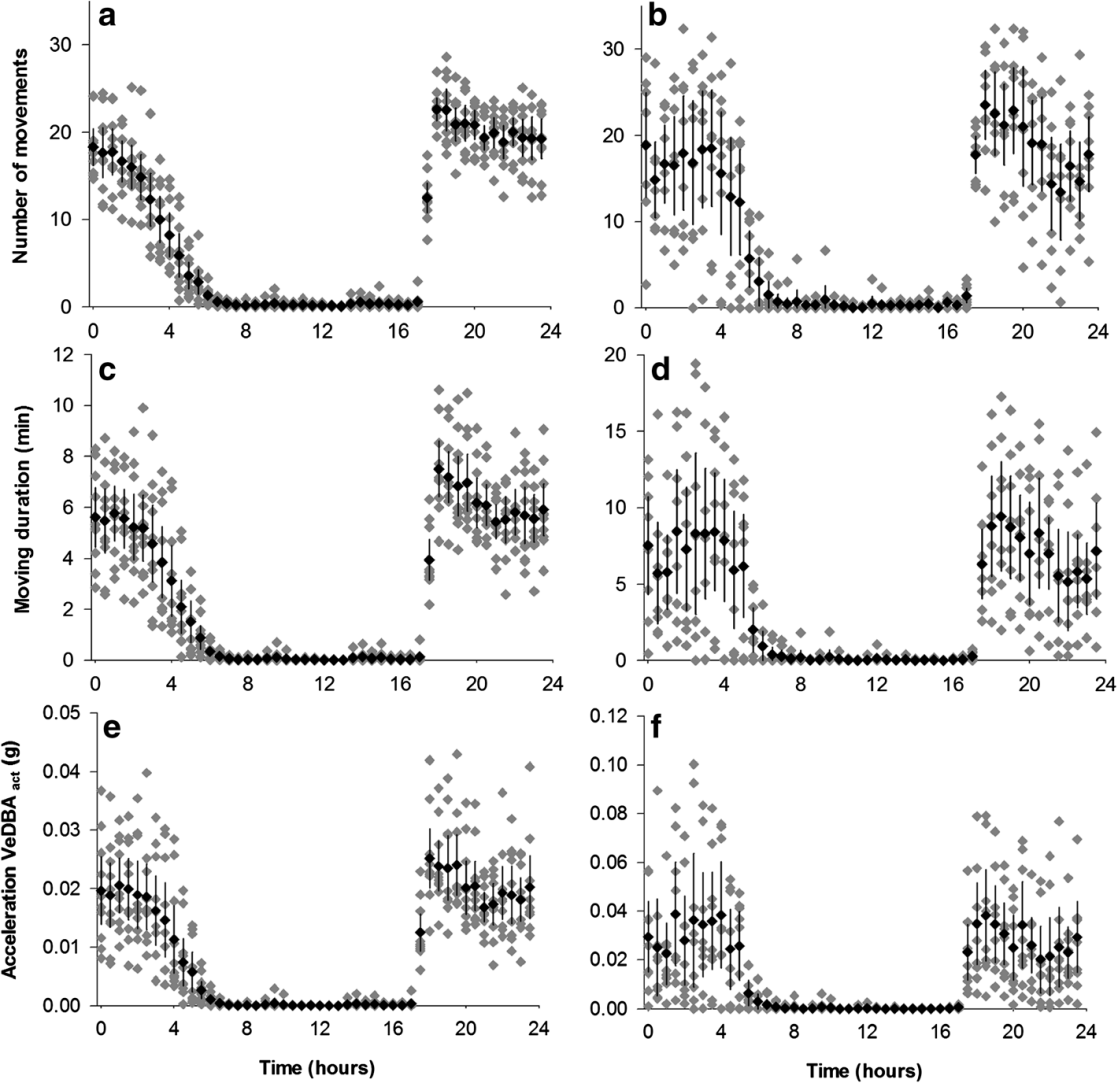
*T. niloticus* activity for 30-min intervals over 24 h. Activity was measured in New Caledonia (**a**, **c**, **e**) and Vanuatu (**b**, **d**, **f**). Grey diamonds, data from each specimen; black diamonds, group average; vertical bars, 95 % confidence interval

Activity patterns calculated over 24 h were homogeneous between specimens (Fig. 6). Activity was nearly non-existent during the day (*N* = 1 ± 1). Topshells began to display activity at 17:30 corresponding to dusk in New Caledonia (*N* = 13 ± 3; D_act_ = 4 ± 1.3 min; VeDBA_act_ = 0.012 ± 0.005 ɡ) and to the first half hour without light in Vanuatu (*N* = 18 ± 3; D_act_ = 6.3 ± 3.7 min; VeDBA_act_ = 0.023 ± 0.016 ɡ). In New Caledonia, activity was intense between 18:00 and 00:00 (*N* = 20 ± 3; D_act_ = 6.2 ± 1.7 min; VeDBA_act_ = 0.02 ± 0.007 ɡ), then slowly decreased until sunrise (Fig. 6). Although the data appeared to be more heterogeneous in Vanuatu, the peak of activity was between 18:00 and 20:00 (*N* = 22 ± 7; D_act_ = 8.4 ± 4.6 min; VeDBA_act_ = 0.03 ± 0.02 ɡ). Despite a slight decline, activity remained high between 20:30 and 04:00, and halted just before dawn (*N* = 16 ± 8; D_act_ = 6.7 ± 4.9 min; VeDBA_act_ = 0.03 ± 0.02 ɡ; Fig. 6).

**Fig. 7:**
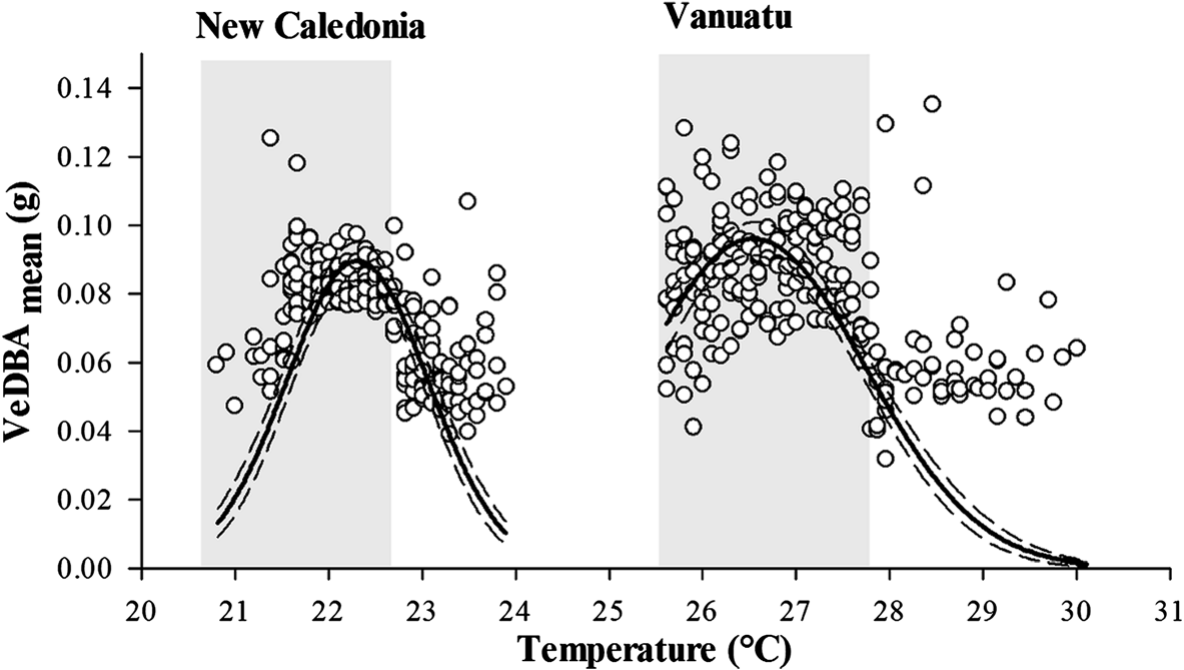
Influence of temperature on *T. niloticus* activity. Activity (VeDBa mean) of topshells in New Caledonia and Vanuatu averaged for each 0.1 °C increment in water temperature. Shaded areas indicate night temperatures for both sites. Black curves correspond t the Gaussian distribution fitted on the data with 95 % confidence intervals

Activity of the topshells, represented by VeDBA _mean_, increased with temperature to 22.3 and 26.5 °C in New Caledonia and Vanuatu, respectively, at which point activity levels declined slightly (Fig. 7, all parameters *P* < 0.001, R^2^ > 0.7). Despite the difference in temperature between the two sites, activity levels are comparable (T-test, *P* = 0.36) and appeared to be associated to light levels (Fig. 7).

### The effect of size on activity

**Fig. 8:**
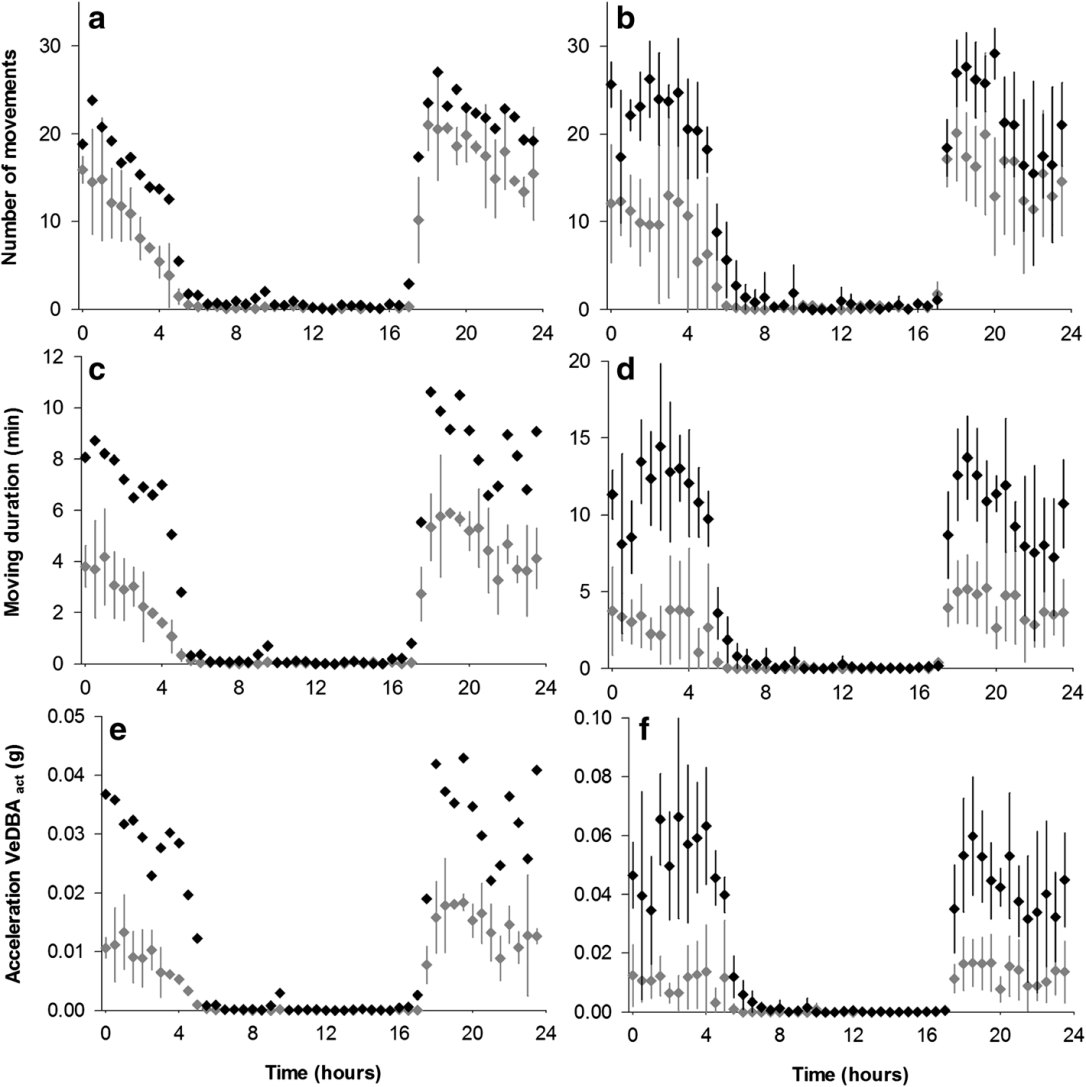
The effect of size on *T. niloticus* activity. Measurements were taken for specimens from New Caledonia (**a**, **c**, **e**) or Vanuatu (parts **b**, **d**, **f**). Activities were determined for large specimens (grey diamonds) and small specimens (black diamonds) and summed for 30-min intervals over a 24-h period. Vertical bars, 95 % confidence interval. In New Caledonia, only three individuals were measured (two large and one small)

In Vanuatu, two set of topshells were observed: four small organisms and four large organisms measuring 85.5 ± 5.1 mm and 132.3 ± 9.2 mm, respectively. These size-based groups did not differ in activity rhythms, but did differ in the activity intensity (Table 1). All the variables used to describe the activity (N, D_act_, VeDBA_act_) showed significantly higher levels for small than for large specimens (Fig. 8, T-test, *P* < 0.001). The activity measured for the two large topshells in New Caledonia have an activity comparable to those of Vanuatu (T-test, *P* > 0.05). The activity of the small specimen in New Caledonia although higher than that of the two large (Fig. 8, T-test, *P* < 0.001), is significantly lower than those of Vanuatu (T-test, *P* < 0.001). The most intense (VeDBA_mean_ > 0.26 ɡ) and longest (D_mov_ > 3.5 min) movements were only performed by small topshells. For the same 0.1 °C increment in water temperature, small specimens showed a significantly higher activity (VeDBA_mean_) than large topshells for both sites (T-test, *P* < 0.001).

## Discussion

### Rhythms of activity

Determining the periods of active behaviour is essential in animal ecology. In this study, we presented one of the first uses of accelerometers on a gastropod, *T. niloticus*, in order to describe the activity rhythms of this organism at two sites (Vanuatu and New Caledonia).

The instrumented topshells clearly displayed activity based on a 24-h cycle, with 95 % of movements made at night. Activity began abruptly around 17:30, regardless of the study site and water temperature. Activity levels were intense until midnight and then slowly decreased until sunrise. This nocturnal activity was previously known due to direct observations and was associated with reproduction [36, 39] as well as feeding; topshells are grazing herbivores and detritivores, but no quantification of this activity has been made [21, 36, 37]. Since the breeding season is between November and May, the movements measured here in June-July and mostly of low intensity (duration <30 s, VeDBA_mean_ <0.12 ɡ) would likely correspond to foraging movements. While the activity start was common to all topshells, the activity stop varied among individuals during the night time. This behaviour could be associated with a state of satiation being reached during the night. This nocturnal behaviour is not unusual and has been observed in other gastropods such as *Gibbula nivosa* [49], *Turbo marmoratus* [50], *Turbo chrysostomus* [51], and *Haliotis asinia* [52], and was always associated with feeding behaviour. To validate this hypothesis, the study of the gut contents of *T. niloticus* during a 24-h cycle could be considered as previously performed on *Haliotis asinia* [52].

On a 24-h cycle, dusk is a period in which algae had maximum nutrients at the end of the photosynthetic period corresponding to profitable conditions for grazers to feed at the end of the light period [53, 54]. Moreover, the concentration of oxygen was maximized [55, 56], and turbulence due to trade winds was low [57] which were favourable conditions for the initiation of topshell activity. For many of the common coral reef herbivorous macroinvertebrates, such as gastropods [51, 58, 59] and small crabs [60], nocturnal feeding activity has been proposed to be a response to predation and competition with grazing fishes [61]. Herbivorous fishes are abundant in New Caledonia [62, 63] and their importance as grazers is well recognized [64].

### Comparisons between study sites and the effect of size

The main difference between the two study sites was the sampling temperatures measured during monitoring. This temperature was on average 22.5 ° C in New Caledonia and 27.3 °C in Vanuatu. It is well known that fundamental physiological functions of ectotherms are particularly influenced by temperature [65–67]. However, the pattern and intensity of the measured activity were comparable between the two sites despite this difference of 5 °C. The voluntary performance of free ranging topshells did not seem to be influenced by environmental temperature. Moreover, since in our study the variations in light and temperature are correlated at both sites, it is difficult to discern whether peaks of activity were a response either factors. Animals usually select temperature ranges that maximize their physiological performance e.g. through optimizing muscle function [68]. In addition, during our study, the tagged topshells did not experience the extreme temperatures that can occur in their environment or within their geographical distribution. Indeed, the temperatures measured in Vanuatu are comparable to those found in New Caledonia during the warm season [40]. Thus, in the present study, we were not able to measure the activity corresponding to the thermal performance curve of this species as reported by Gannon et al. [48].

During monitoring, two size classes were identified: small (B_d_ < 100 mm) and large topshells (B_d_ > 100 mm) showing comparable activity patterns. Large specimens had similar activity (duration, number of movement, acceleration) regardless of the study site and water temperature. In both sites, small topshells exhibited a higher activity with more intense movements than large ones. Moreover, the small topshells from Vanuatu have also depicted a higher activity than the small specimen from New Caledonia. These results were comparable to the measurements of respiration made by Lorrain et al. [40] on topshells from New Caledonia during the warm and cool seasons. Indeed, the large specimens had similar respiration rates during the night between the warm and cool seasons (9.9 vs 8.6 μmol.C.g^−1^). Small trochus had respiration rates higher than large specimens with a maximum during the warm season (17.6 vs 12.7 μmol.C.g^−1^). Previous studies have demonstrated that oxygen consumption correlates with temperature [40, 68, 69]. Body size would be one of the most important factors affecting metabolism. Indeed, the ratio between the gas exchange surface for respiration and the body volume decreases with growth. Thus, per gram of dry body weight, oxygen consumption is higher among younger, small individuals than in adults [70–72].

The activity observed for small topshells was characterized by longer and more intense movements. This activity could be associated with topshells moving longer distances during the night. During the present monitoring in Vanuatu, the location of each individual was identified in relation to 3 fixed poles (used as landmarks) every 90 min. Small topshells travelled an average distance of 26.3 ± 5.3 m against 11.5 ± 2.6 m for large specimens during 71 h of monitoring. Moreover, smaller specimens clearly migrated to the reef edge, while the four largest specimens remained in the tracking area (Dumas et al., in prep.). Byers [73] previously showed that for estuarine snails, although the amount of food was sufficient, smaller snails always dispersed at relatively higher rates than larger specimens. These intense movements may correspond to migration/dispersion from the intertidal reef flat to outer reef crests [28, 31–33]. These movements may also be associated with occasional trips related to the local microhabitat. *T. niloticus* can move quickly when small-scale biotic/abiotic factors, such as hydrodynamics, nature of the substrate, and food availability, do not suit them [35].

### Strengths and limitations of the present study

The use of HOBO accelerometers yielded many advantages. They were easy to use, data were downloadable daily while specimens remained underwater, and the files were small and easy to handle. The monitoring period was only limited by the accelerometers’ battery life and the diver’s availability to visit the sites each day. However, this type of accelerometer also has limitations, particularly with regard to sampling frequency. The minimum programmable frequency of the device is 0.25 Hz which corresponds to one measurement every 4 s. In the present study, this frequency was high enough to describe activity patterns of topshells but this frequency range excludes motions with a duration less than 4 s and induces inaccuracies in the characterization of the movements. In the context of future bioenergetic studies dedicated to the study of the oxygen consumption versus activity, it would be preferable to increase the frequency of acquisition. In addition, this sampling time step was insufficient to estimate the distance travelled by the topshells during the monitoring period. Indeed, estimating speed and distance travelled from the acceleration data is theoretically possible [74], but doing so amplifies measurements errors producing unreliable predictions of future location [75]. Finally, combining these accelerometers with a current sensor and video recordings allow us to further characterise of the types of topshell movements, including locomotion, feeding, escape, or defence against predators. In particular, calibrating movements according to body angles, pitch, and roll [11, 19] is useful in the classification of animal movement patterns [12], as any body posture will narrow the range of possible behaviours or be characteristic of one behavioural pattern for the study animal.

## Conclusions

Combined with other instruments, accelerometers can provide a wide range of detailed information on the environmental context of animal behaviour and physiology that can exceed the descriptive abilities of the human observer, particularly during cryptic periods. The ability to identify locomotion has broad ecological applications. For instance, temporal patterns in locomotion can provide insights, complementary to direct observations and experimental studies, into changes in behavioural rhythms such as those that occur during the onset of migration [20, 39, 76, 77] or reproduction [36, 39] and in response to environmental change [13]. Such information can be applied to specific conservation problems for *T. niloticus.* For example, at the level of individual topshells, monitoring the behaviour of captive-bred individuals after release, particularly individuals <60 mm [30, 78–80] may be useful. Monitoring may be scaled up to estimate area use at the population level through multiple device deployments, to assess behavioural responses to changes in the environment, including restocking [81] and establishing marine protected areas [27], and to assess the exploitation of this species.
